# Effect of Incision Negative Pressure Wound Therapy on Donor Site Morbidity in Breast Reconstruction with Deep Inferior Epigastric Artery Perforator Flap

**DOI:** 10.1016/j.jpra.2022.08.002

**Published:** 2022-08-18

**Authors:** Songsu Kang, Seiko Okumura, Yoko Maruyama, Ikuo Hyodo, Ryota Nakamura, Saya Kobayashi, Maho Kato, Keisuke Takanari

**Affiliations:** a Department of Plastic and Reconstructive Surgery, Aichi Cancer Center Hospital, Aichi, Japan; b Department of Plastic and Reconstructive Surgery, University of Occupational and Environmental Health, Fukuoka, Japan

**Keywords:** Closed incision negative pressure wound therapy, Breast reconstruction, Deep inferior epigastric artery perforator flap, Postoperative wound complication

## Abstract

**Background:**

The usefulness of closed incision negative pressure wound therapy (ciNPWT) has been well documented in many surgical sites, except for the donor site of the deep inferior epigastric artery perforator (DIEP) flap. The aim of this study was to evaluate the effect of ciNPWT on microsurgical breast reconstruction using a DIEP flap.

**Methods:**

Fifty-six cases of breast reconstruction with DIEP flap were included and divided into two groups based on post-surgical wound management: the ciNPWT group received ciNPWT at the donor site, while the conventional group received conventional wound management. The primary outcomes were the incidence of seroma, wound dehiscence, and surgical site infection, and secondary outcomes were the time to drain removal and amount of drainage. The breast reconstruction risk assessment (BRA) score was used to evaluate the comprehensive risk in each case.

**Results:**

Among the patient and surgical characteristics, only the BRA score (P=0.02) and the time to elevate the flap (P=0.02) were significantly higher and longer in the ciNPWT group, respectively. The incidence of seroma, dehiscence, and wound infection showed no significant difference between the two groups. In the subgroup analysis of patients with body mass index ≥ 25, the primary outcomes did not differ, while the secondary outcomes were significantly lower in the ciNPWT group (drainage volume, P = 0.04; time to drain removal, P = 0.04)

**Conclusion:**

ciNPWT can potentially reduce the incidence of donor site complications of DIEP flaps, especially if the comprehensive risk for post-surgical complications is considered.

## Introduction

The free transverse rectus abdominis musculocutaneous (TRAM) flap is a useful method for breast reconstruction and can be used for reconstruction of a large and drooping breast. Currently, free TRAM flaps are considered reliable, with success rates approaching 100% along with good cosmetic results and low functional abdominal morbidity.^1^ Advances in microsurgery, mature surgical procedures, establishment of the angiosome theory, and improvements in operative devices have increased the flap engraftment rates, while the muscle-sparing harvest technique for deep inferior epigastric artery perforator (DIEP) flap has made it possible to minimize the damage to abdominal function.^2^

Negative pressure wound therapy for closed surgical incision (ciNPWT) is widely recognized as an adjunct therapy to reduce postoperative complications.^3^, ^4^, ^5^ The World Union of Wound Healing Societies (WUWHS) recommends applying ciNPWT to patients with high-risk factors for surgical site complications.^6^ WUWHS also proposes that indications for ciNPWT should be based on the risk factors associated with patient characteristics and the surgical procedure.

Breast reconstruction using autologous tissue is considered a surgical procedure with a low incidence and lower consequences of surgical site complications. Therefore, there are few reports on the effectiveness of ciNPWT for microsurgical breast reconstruction, and even fewer reports on its effect on the donor site. However, in breast reconstruction as an elective cosmetic surgery, problems at the donor site need to be avoided, and prevention of donor site morbidity is still an issue which needs to be resolved. In this study, we investigated the usefulness of implementing ciNPWT in the donor part of a free DIEP flap.

## Material and Methods

### Patients and Outcomes

This retrospective study was conducted in a single institution, following approval from the institutional ethical review board. All patients provided their written consent to this study. From June 2018 to May 2020, a series of 56 consecutive patients who underwent breast reconstruction with a free DIEP flap at Aichi Cancer Center Hospital were included. The patients were divided into two groups: in one group, ciNPWT was applied for post-surgical wound management (ciNPWT group), while in the other group, conventional wound management was applied with film dressing (conventional group). To avoid a selection bias, ciNPWT was applied in all cases who underwent breast reconstruction with a DIEP flap from August 2019 to May 2020, from when ciNPWT was introduced in our institution. As a control group of the same size, we included consecutive patients retrospectively who underwent the same procedure.

Data collection was performed in a retrospective fashion using patient charts and data on patient age, body mass index (BMI), administration of neoadjuvant chemotherapy, smoking history, history of diabetes mellitus, history of previous abdominal operation, the American Society of Anesthesiologists- physical status (ASA-PS) and breast reconstruction risk assessment (BRA) scores (reference), as well as operative characteristics that included immediate or delayed reconstruction, weight of the resected mammary gland, flap size (vertical and horizontal), number of perforators, and time to flap harvest were collected. The primary outcomes were the incidence of postoperative seroma, wound dehiscence, and surgical site infection, while the secondary outcomes were the time to drain removal and amount of drainage.

### Surgical Technique and Postoperative Wound Management

All flaps were elevated with a DIEP flap containing multiple perforators. After flap harvest, the anterior layer of the rectus sheath was closed with interrupted sutures using absorbable threads. A suction drain was placed on the fascia. The donor site was closed in three layers, with the superficial fascia and subcutaneous layer closed with buried sutures, and the skin with continuous sutures. No other means for seroma prevention were used, such as quilting suture, tissue adhesive glue, or local steroid administration.

**Fig. 1:**
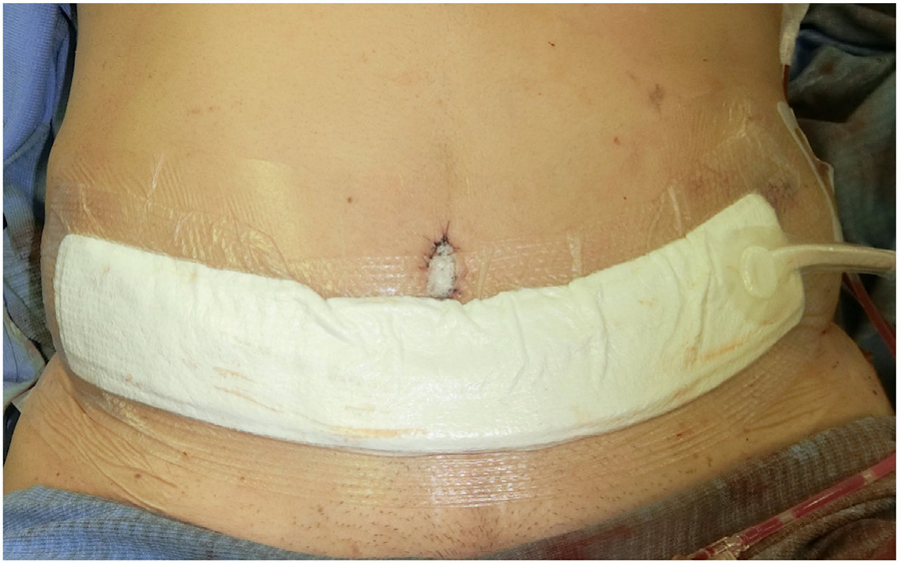
Intraoperative view of ciNPWT in this study. The foam is directly attached on the wound. When the foam dressing does not reach to cover full length of the wound, film dressing is used to cover the lateral ends of the wound.

CiNPWT was performed using the PICO® single-use portable negative pressure wound therapy system (Smith &Nephew plc, Watford, England, UK). The size of the foam dressing was 10 cm × 30 cm in all cases (Fig. 1). CiNPWT was applied until postoperative day 10, and the foam dressing was replaced when the dermal stitches were removed on the 6th day after the operation. After detachment of the PICO on postoperative day 10, alprostadil alfadex ointment was used for wound treatment. In the control group, a film dressing was used for wound management until postoperative day 6 when the dermal stitches were removed, and the aforementioned ointment was used until the closed incision had healed.

### Definition of Donor Site Morbidity

Seroma was defined as a fluid collection requiring any drainage procedures such as aspiration, drain insertion, or surgical capsulotomy. The timing of the drain removal was determined according to the criteria listed in Table 1. Wound dehiscence was defined as splitting apart of the closed wound from the margins along some or all of its length. Dehiscence was classified into superficial or deep, depending on whether it was at the skin level or below the subcutaneous tissue. Wound infections were diagnosed based on clinical findings and included those that required drainage and/or antibiotic administration in addition to routine postoperative care.

**Table 1:** Criteria for Drain Removal

Consider removal with a drainage volume less than 30 ml/day.
Set 14 days after operation as a guide for maximum placement.
Immediately remove when suspected drain related infection.

### Statistical Analysis

Univariate analysis was performed using Mann-Whitney rank sum test to compare groups that were non-parametric, while Welch's t-test was used to analyze if two populations had equal means. The chi-square test was used for evaluating relationships between categorical variables, and Fisher's exact test for evaluating relationships between categorical variables in smaller numbers (<10). Analyses were performed using STATA version 16.0 (Stata Corp, College Station, TX, USA).

## Results

### CiNPWT Group Vs Conventional Group, As a Whole

The patient background and intraoperative characteristics are shown in Table 2. In the ciNPWT group, the BRA score was significantly higher (P=0.02) than that in the conventional group, indicating that the risk of complications was higher in the ciNPWT group. No other variables among the patient demographics were significantly different between the two groups. Although BMI tended to be higher in the ciNPWT group (P=0.08), the proportion of pre-obese and obese patients (BMI ≥ 25) was not statistically different between the groups. The time to elevate the flap was significantly longer in the ciNPWT group (P=0.02). Additionally, the horizontal length of the flap and the number of perforators tended to be larger in the ciNPWT group (length of flap, P=0.08; number of perforators, P=0.06). The other operative factors did not show any difference between the two groups.

**Table 2:** Demographics of all patients

		ciNPWT (n=28)	Conventional (n=28)	P value
**Patient characteristics**				
Age, mean (SD)		48.9 (6.9)	51.6 (9.1)	0.22
Body mass index,mean (SD)		25.9 (4.7)	23.9 (3.7)	0.08
≥25, n (%)		14 (50%)	10 (35%)	0.28
NAC, n (%)		9 (32%)	8 (29%)	0.77
smoke, n (%)		1 (3.6%)	2 (7.1%)	1.00
Diabetic mellitus, n		0	0	-
pre-ope, n (%)		12 (43%)	10 (36%)	0.58
ASA-PS, n (%)	I	13 (46%)	11 (39%)	0.58
	II	15 (54%)	17 (61%)	
	≥III	0 (0%)	0 (0%)	
BRA score*, median		19.7	17.2	0.02
(range)		(13.2-40.3)	(13.7-24.8)	
**Operative characteristics**				
immediate reconstruction, n (%)		20 (71%)	23 (82%)	0.34
weight of resected mammary gland, mean (SD)		410 (174)	394 (203)	0.76
flap size, mean (SD)	vertical	12.9 (0.95)	12.4 (1.1)	0.10
	horizontal	31.1 (4.4)	29.4 (2.3)	0.08
number of perforators, mean (SD)		6.4 (1.6)	5.6 (1.3)	0.06
time to flap harvest, mean (SD)		285 (43)	258 (38)	0.02

Abbreviations: SD, Standard deviation; NAC, neoadjuvant chemotherapy; pre-ope, history of previous operation on abdomen; ASA PS, American Society of Anesthesiologists physical status

⁎ BRA score, means predicted probability of overall complications

There were no donor-site complications resulting in reoperation in either group (Table 3). The incidence of postoperative seroma was not significantly different between the two groups. Among cases with development of seroma, additional statistical analysis was conducted on the number of aspirations, amount of aspiration volume, and period until final aspiration. However, no difference between the two groups was found for any of these items. Similarly, there was no difference with regard to wound infection and wound dehiscence between the two groups. Although, the drain indwelling period tended to be lower in the ciNPWT group, the difference was not so statistically significant (P=0.06).

**Table 3:** Outcomes in all patients

	ciNPWT (n=28)	Conventional (n=28)	P value
**primary outcome**			
seroma, n (%)	13 (46%)	13 (46%)	1.00
number of aspirations, mean	3	3.5	0.75
total drained volume, mean	161	134	0.62
period for depletion, mean	34	31.5	0.78
infection, n (%)	6 (21%)	5 (18%)	0.73
dehiscence, n (%)	3 (11%)	4 (14%)	1.00
**secondary outcome**			
drainage period, median	10	13	0.06
(range)	(6−14)	(8−19)	
drainage volume, median	557	638	0.31
(range)	(129-1422)	(232-2763)	

### Subgroup Analysis for Patients with BMI≥25

We further performed subgroup analysis for patients with a BMI ≥ 25. In this subgroup, there was no difference in the patient and surgical background between the ciNPWT and conventional groups (Table 4). Regarding the primary outcomes, no significant difference was found in the incidence of seroma, wound infection, or wound dehiscence. However, when evaluating the secondary outcomes, the drainage volume (P = 0.04) and time to drain removal (P =0.04) were significantly lower in the ciNPWT group (Table 5).

**Table 4:** Demographics of patients with BMI ≥25

		ciNPWT (n=14)	Conventional (n=10)	P value
**Patient characteristics**				
Age, mean (SD)		49.2 (7.7)	55.5 (6.5)	0.05
Body mass index, mean (SD)		29.4 (3.9)	28.2 (2.3)	0.36
NAC, n (%)		3 (21%)	3 (30%)	0.67
smoke, n (%)		1 (7.1%)	0 (0%)	1.00
Diabetic mellitus, n		0	0	-
pre-ope, n (%)		6 (43%)	4 (40%)	1.00
ASA-PS, n (%)	I	6 (43%)	1 (10%)	0.17
	II	8 (57%)	9 (90%)	
	≥III	0 (0%)	0 (0%)	
BRA score, median		23.0	21.0	0.11
(range)		(17.0-40.3)	(18.5-24.8)	
**Operative characteristics**				
immediate reconstruction, n (%)		9 (64%)	6 (60%)	1.00
weight of resected mammary gland, mean (SD)		512 (189)	534 (243)	0.83
flap size, mean (SD)	vertical	13.6 (0.56)	12.9 (1.2)	0.19
	horizontal	33.4 (4.6)	28.0 (1.8)	0.09
number of perforators, mean (SD)		6.7 (1.8)	5.5 (1.0)	0.05
time to flap harvest, mean (SD)		292 (40)	259 (38)	0.06

**Table 5:** Outcomes in patients with BMI ≥25

	ciNPWT (n=14)	Conventional (n=10)	P value
**primary outcome**			
seroma, n (%)	6 (43%)	6 (60%)	0.41
infection, n (%)	5 (36%)	3 (30%)	1.00
dehiscence, n (%)	1 (7.1%)	1 (10%)	1.00
**secondary outcome**			
drainage period, median	10	13	0.04
(range)	(7−14)	(8−19)	
drainage volume, median	528	980	0.04
(range)	(302-1422)	(232-2763)	

## Discussion

The free TRAM flap, which consists of soft natural tissue and an abundant vascular network, is the most popular technique for breast reconstruction and is most suitable for large and/or drooping breasts. Since the success rate of free tissue transfer has increased, microsurgical breast reconstruction is no longer considered a hesitant procedure, but rather the most reliable procedure for achieving natural esthetic results.^1^ Furthermore, the TRAM flap modified as the muscle-sparing TRAM (MS-TRAM) or DIEP flap is associated with lower donor-site morbidity due to less harvesting of muscle and anterior rectus fascia.^2^^,^^7^, ^8^ The muscle-sparing harvest technique minimizes abdominal wall dysfunction and leads to a lower incidence of abdominal bulge/hernia.

While the MS-TRAM and DIEP flaps have been optimized for achieving both esthetic and functional outcomes with microsurgical stability,^9^, ^10^ some challenges remain. In most studies, there has been more focus on the esthetic outcomes of the reconstructed breast and less on donor-site complications such as seroma, wound dehiscence, and wound infections, although these can lead to patient dissatisfaction.^11^ Considering that breast reconstruction is an elective surgery with the purpose of esthetic improvement and patient satisfaction, donor-site complications should be avoided as much as possible, and surgeons should focus on their prevention.

Many reports on multiple surgical sites refer to ciNPWT as a useful adjunctive therapy to reduce the incidence of surgical site complications. In total hip or knee arthroplasty, several meta-analyses of randomized controlled trials reported that ciNPWT decreased the surgical site infection (SSI) rates, specifically in revision arthroplasty and high-risk patients.^12^^,^^13^ In cardiothoracic surgery, single-use NPWT has been shown to be cost-effective in addition to having a positive impact on reducing sternal wound infection and shortening the length of hospital stay (LOS) in high-risk patients who develop sternal wound infection.^14^^,^^15^ Although many reports have revealed the favorable effects of ciNPWT with respect to SSI or LOS, evidence in breast reconstruction surgery with autologous tissue, especially for the donor site, is limited. In their consensus document, WUWHS recommends that ciNPWT should be considered based on procedure- and patient-related risk factors. According to this document, implant surgery and maxillo-craniofacial pediatric plastic surgery are classified as procedures with lower incidence and severity of surgical site complications, while reduction mammoplasty is associated with a higher incidence and lower severity in relation to plastic surgery. The BRA score is also one of the listed additional risk factors for breast reconstruction according to the WUWHS. The BRA score, proposed by John et al., is an online risk calculator based on the Tracking Operations and Outcomes for Plastic Surgeons (TOPS) program.^16^ It predicts the probability of complications based on patient characteristics including BMI, underlying disease, smoking status, history of chemotherapy and radiation therapy, and suggests the possible application of ciNPWT in relation to patient-related factors.

Considering the issues mentioned above, ciNPWT for plastic surgery should be applied to abdominoplasty, which is considered a relatively high-risk surgery for postoperative complications. There are a few reports on the usefulness of ciNPWT in the donor site of breast reconstruction surgery with a free abdominally based flap. Muller et al. reported that the frequency of dehiscence in the donor-site was significantly lower in the ciNPWT group after analyzing 51 microsurgical breast reconstructions using the DIEP or profunda artery perforator (PAP) flap.^17^ Laura et al. reported that in 225 cases of breast reconstruction with abdominal free flap, ciNPWT significantly reduced the number of overall and operative complications.^18^ However, each outcomes’ wound dehiscence, seroma and wound infection did not show any difference. Wang et al. also researched their 126 cases of breast reconstruction with abdominal free flap and showed inferiority of ciNPWT in LOS.^19^ In these reports, the surgical procedure―DIEP or PAP flap in the former, MS-TRAM or DIEP for bilateral or unilateral reconstruction in the latter two―is not unified, which is thought to affect the incidence of post-surgical complications. Therefore, confounding bias can easily interfere with the examination results. Moreover, since their selection of patients to apply ciNPWT was arbitrary, a higher risk of selection bias was involved and can distort the interpretation of the results.

In our study, we have made some efforts to eliminate these biases. First, to minimize the confounding effects relating the surgical procedure, the flap harvest technique was homogeneous, and only a unilateral DIEP flap was included. Second, to avoid selection bias, we assigned the patients into the ciNPWT group or the conventional group based not on physician's decision but serial selection. Another notable point for our study is that we used the BRA score, which utilizes comprehensive patient-related risk factors, in order to understand whether the effect of ciNPWT correlated with the objective risk index.

The present study showed no statistical difference between the two groups with regard to any of the complications, which is similar to the preliminary studies by Laura et al. and Wang et al. This is owing to small sample size and lower incidence of complications. However, the BMI tended to be higher in the ciNPWT group, with the BRA score being significantly higher. Remarkably, the other patient characteristics showed no statistical differences. This indicates that the ciNPWT group included patients with a higher risk than those in the conventional group. Intraoperative factors in the ciNPWT group also showed a trend toward a statistically significant increase in the risk, number of perforators, flap length, and flap elevation time. When considering the higher risks associated with both patient- and procedure-related factors, the ciNPWT group demonstrated a lower than expected incidence of donor-site morbidity, which was accompanied by a downward trend in the drainage period, even though a statistically significant difference was not clear at this time.

On the other hand, in patients with BMI ≥ 25, there was no difference between the two groups in the patient background, including BRA score and intraoperative factors. In the subgroup comparison, the volume and drainage period were significantly reduced in the ciNPWT group. These findings revealed the effectiveness of ciNPWT in reducing the donor site complication rate more clearly in groups with matched patient background and has significant meaning that ciNPWT has a reducing effect for the volume and drainage period in addition to reported effect. Moreover, ciNPWT may reduce the need for prophylactic procedures such as quilting suture or progressive tension suture in addition to prevention of SSI.

We used PICO® single-use portable negative pressure wound therapy system (Smith &Nephew plc, Watford, England, UK) instead of PREVENA^TM^ Incision Management System (KCI, San Antonio, TX, USA) which was more commonly adopted for ciNPWT in many previous reports. We think PICO® has advantages in its portability and cost compared to PREVENA^TM^. Since PICO® is pocket-sized and smaller than PREVENA^TM^, patients are free from inconvenience in postoperative rehabilitation. From the standpoint of unit price, PICO® can save the cost by $73.8 compared to PREVENA^TM^. On the other hand, PREVENA^TM^ can be applied to more complex shaped wounds by using customizable foam and demonstrate compression effect by more powerful pressure than PICO®. Although PREVENA^TM^ can be a good candidate, we value the advantage of PICO® and choose it for ciNPWT.

There are some limitations to this study. First, some bias could not be avoided due to the retrospective research design, such as an information bias because of the possibility of inaccurate data collection. Moreover, although deviations in patient and surgical characteristics that could be confounding were observed, they could not be statistically controlled. In addition, the sample size may have insufficient statistical power for detecting the “true” effect of ciNPWT. The second limitation concerns the BRA score. There is controversy regarding the usage of the BRA score in patients with different ethnic backgrounds, because this score is based on the Western population. Bloo et al. reported ethnic differences in mortality, length of hospital or intensive care unit stay, and surgical complications.^20^ For instance, the occurrence of hypertrophic scar, a well-known morbidity in plastic surgery, is affected by racial background.^21^ Regarding breast reconstruction with abdominal flaps, Asians, who tend to have less redundant abdominal tissue than Western people, may experience wound tension after donor closure.^22^

In the future, a prospective randomized control study will be required to assess the prophylactic effect of ciNPWT for abdominal flap-based breast reconstruction. In addition, the true endpoint, cost-effectiveness, length of hospital stay, and patient satisfaction should be examined in relation to ciNPWT.

## Conclusion

In conclusion, our study demonstrated that the incidence of wound-related complications did not increase despite the high risk observed in the ciNPWT group. Furthermore, in the subgroup analysis in patients with BMI≥25, a lower incidence of complications was observed in the ciNPWT group. We believe that ciNPWT for DIEP flap donor sites may help to achieve higher patient satisfaction in breast reconstruction.

## Declaration of Competing Interest

None.
